# Changes in tongue–palatal contact during swallowing in patients with skeletal mandibular prognathism after orthognathic surgery

**DOI:** 10.1371/journal.pone.0251759

**Published:** 2021-05-19

**Authors:** Haruka Kagawa, Masato Kaku, Taeko Yamamoto, Yuka Yashima, Hiromi Sumi, Takashi Kamiya, Ichiro Yamamoto, Kotaro Tanimoto

**Affiliations:** 1 Department of Orthodontics, Applied Life Sciences, Hiroshima University Institute of Biomedical & Health Sciences, Hiroshima, Japan; 2 Division of Oral Health Sciences, Department of Anatomy and Functional Restorations, Hiroshima University Graduate School of Biomedical and Health Sciences, Hiroshima, Japan; 3 Kamiya Orthodontics & Dental Clinic, Hamamatsu, Japan; 4 EPG Research Center, Yamamoto Dental Clinic, Hyogo, Japan; Sapienza University of Rome, ITALY

## Abstract

This study aimed to evaluate improvement of tongue-palatal contact patterns during swallowing after orthognathic surgery in mandibular prognathism patients. Thirty patients with mandibular prognathism treated by orthognathic surgery (average age of 27 years, 3 months) and 10 controls (average age 29 years, 6 months) participated in this study. Tongue-palatal contact patterns of patients before and three months after surgery were evaluated by electropalatography (EPG) as well as controls. Whole total of tongue-palatal contact at 0.3, 0.2, and 0.1 sec before complete tongue-palatal contact during swallowing were evaluated. The duration of swallowing phases was also examined. Complete contact of tongue-tip in the alveolar part of individual artificial EPG plate were shown at 0.3, 0.2, and 0.1 sec before complete tongue-palatal contact in the controls, although incomplete contact in the alveolar part were shown at 0.3 sec in mandibular prognathism patients. Whole total of tongue-palatal contact at 0.3 and 0.2 sec before complete tongue-palatal contact was significantly lower in the patients before surgery than in the controls (*p*<0.05). However, these values increased after surgery. The duration of oral and pharyngeal phase was significantly longer in the patients before surgery than in the controls and the patients after surgery (*p*<0.01). This study demonstrated that the tongue-palatal contact pattern improved and the duration of oral and pharyngeal phase was shortened in mandibular prognathism patients during swallowing after orthognathic surgery. It is suggested that changes in maxillofacial morphology by orthognathic surgery can induce normal tongue movement during swallowing. (The data underlying this study have been uploaded to figshare and are accessible using the following DOI: https://doi.org/10.6084/m9.figshare.14101616.v1)

## Introduction

Tongue and perioral muscle function are important because they are closely related to dentoalveolar and craniofacial morphology and development [1]. It is well known that abnormal tongue movement and posture during swallowing may cause malocclusion [2, 3]. Therefore, improvement of abnormal tongue function in patients with malocclusion such as skeletal mandibular prognathism is crucial to plan out orthodontic treatment and ensure posttreatment occlusal stability. For severe mandibular prognathism patients, orthognathic surgery is applied to correct upper and lower jaw relationship. Given that orthognathic surgery can generate dynamic alterations in the relationship between the maxilla and the mandible, it is expected that tongue and perioral muscle function will also change after surgery. However, little information is available on the changes in tongue movement by orthognathic surgery during swallowing due to difficulty in visualizing the tongue from outside the oral cavity. Sakaue et al. reported that there is a difference in tongue pressure production between normal subjects and patients with mandibular prognathism during swallowing by a palatal sensor sheet [4]. Using real-time magnetic resonance imaging (MRI) Görgülü et al. showed that the manner of bolus transfer in patients with skeletal Class III malocclusion differed from that of patients with skeletal Class I malocclusion [5]. However, tongue movement cannot be assessed by a tongue pressure measuring device, and it is difficult to observe the position of the tongue on a horizontal projection surface using MRI.

Electropalatography (EPG) is a portable device composed of an acrylic palatal base plate with 62 electrodes and an electrically connected personal computer, which permits the visualization of tongue-palatal contact during speech [6, 7]. Recently, Kojima et al. reported that tongue-palatal contact patterns for /t/ and /s/ articulation improved clearly after SSRO in patients with mandibular prognathism by EPG study [8, 9]. Ichida et al. showed that tongue-palatal contact duration associated with swallowing was closely related to the patient’s maxillofacial morphology by EPG study [10]. Thus, EPG is a variable device that can be used not only for articulation training but equally for the evaluation of tongue movement during swallowing and speech. However, changes in tongue-palatal contact during swallowing in patients with mandibular prognathism before and after orthognathic surgery have not yet been examined. Therefore, in this study, we investigated the effect of orthognathic surgery on tongue-palatal contact patterns and the duration of the swallowing phase in patients with mandibular prognathism by EPG.

## Materials and methods

### Participants

The participants were 30 patients with mandibular prognathism: 8 males and 22 females with an average age of 27 years, 3 months. All participants showed skeletal Class III malocclusion with anterior cross-bite (average ANB angle, − 2.2 °; overjet, − 5.0 mm) and underwent sagittal split ramus osteotomy (SSRO) for setback of the mandible (including 7 patients who underwent Le Fort I osteotomy as well as SSRO). No patients had difficulty in swallowing, but they complained of difficulty in mastication. Severe vertical open bite case was excluded. Ten adults without swallowing problems including tension around the lips or functional dysphagia served as controls (3 males and 7 females; average age 29 years, 6 months). They had skeletal Class I jaw relationship and normal occlusion, and did not receive orthodontic treatment (average ANB angle, 3.2 °; and overjet, 2.8 mm). The present study was conducted in accordance with the guidelines outlined in the Declaration of Helsinki and was approved by the Hiroshima University Hospital IRB, and all participants signed an informed consent agreement (E-476). Written informed consent was obtained from all participants before the initiation of treatment. After orthognathic surgery, we recommended patients to take soft diet in order to assist recovery from jaw surgery for 4 weeks.

### Instrumentation

**Fig 1:**
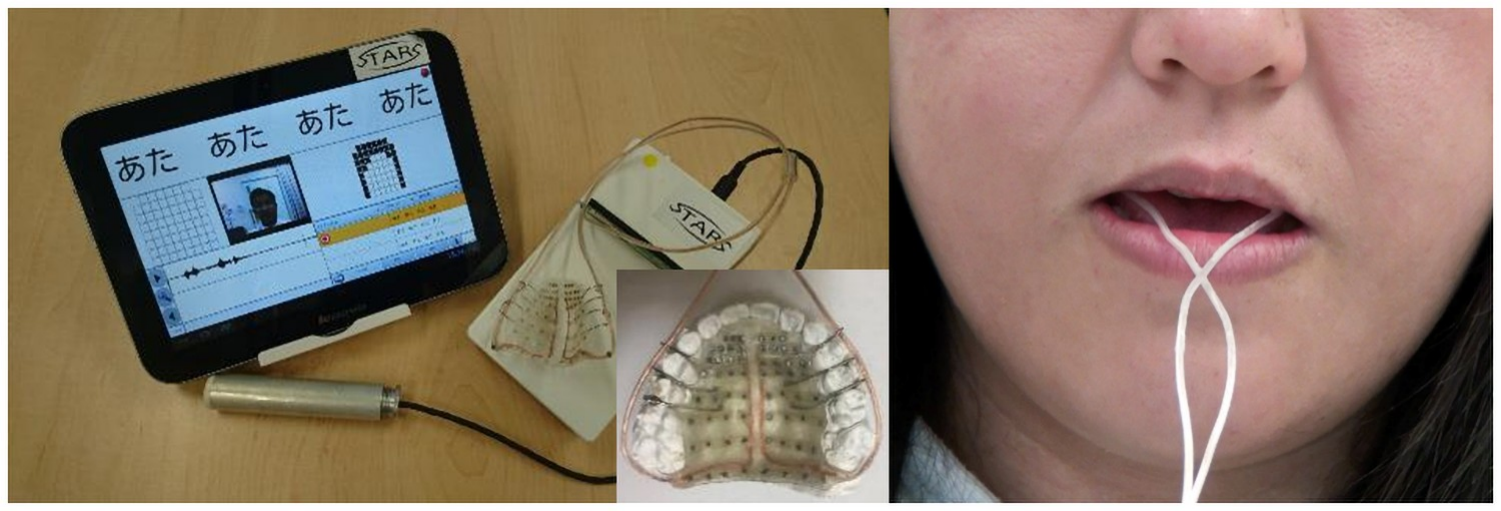
Instrumentation. A tablet-type EPG system and recording system (STARS), and a picture of a participant wearing EPG plate.

**Fig 2:**
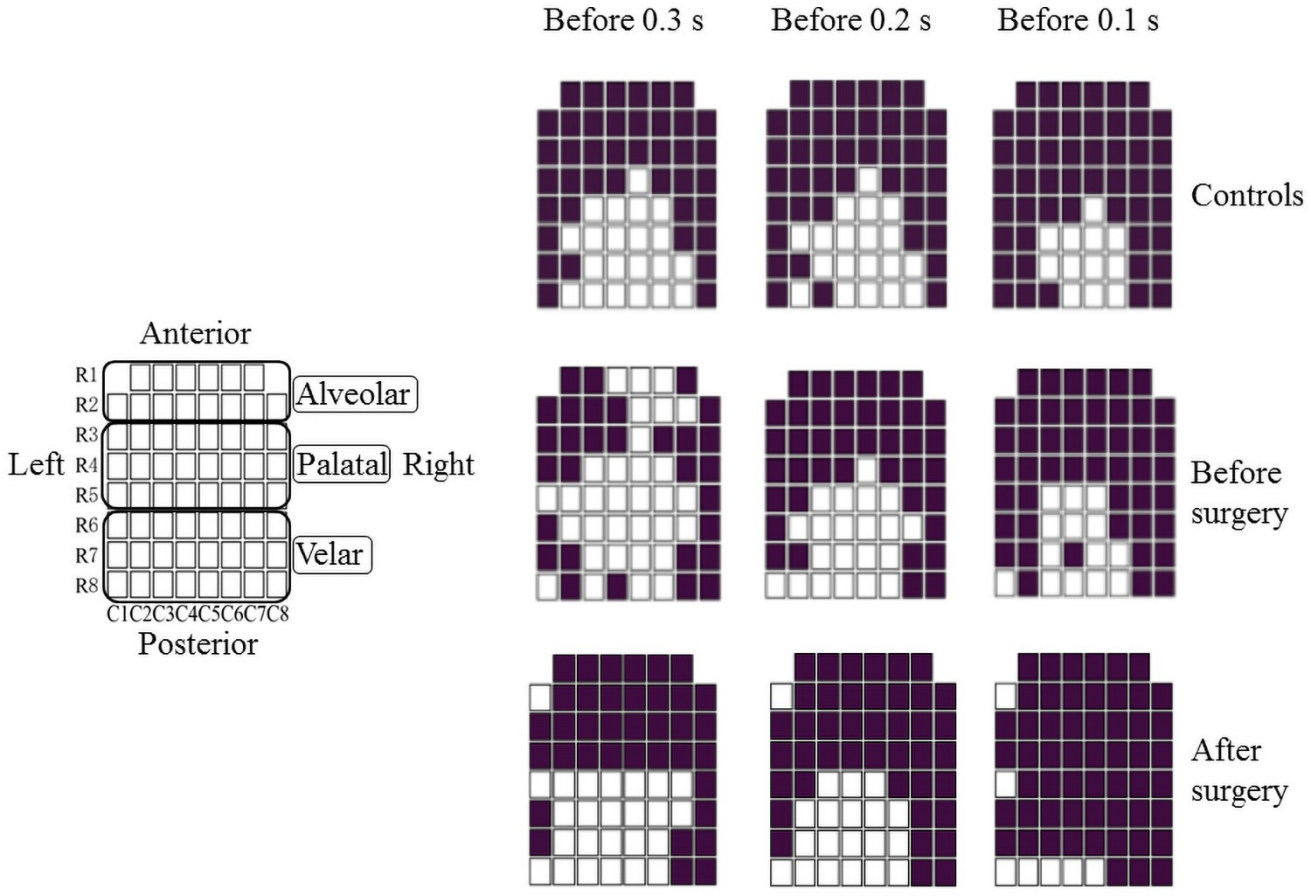
Plate classification. A schematic diagram of electrodes which were segmented by 8 horizontal rows (R1-R8) and vertical columns (C1-C8) across the palate. An EPG plate was divided into 3 parts of mesiodistal direction for detailed analysis (Alveolar, Palatal and Velar). Representative EPG pattern during swallowing in the controls, patients before surgery, and after surgery at 0.3, 0.2 and 0.1 seconds before complete tongue-palatal contact.

A tablet-type EPG system, Speech Training Aid and Recording System (STARS) (ASAHI ROENTGEN Ind. Co., Ltd, Kyoto, Japan) was used in this study (Fig 1). Each participant used an individual artificial EPG plate that was constructed to fit against the hard palate to record the tongue-palatal contact patterns. The EPG plate had 62 silver electrodes lined up according to anatomical landmarks, and a tongue-palatal contact signal was transmitted to the EPG system. The plate equipped with wire connections that passed outside of dental arch in order not to disturb tongue movement during swallowing (Fig 1). The EPG flame was segmented into eight horizontal rows (R1-R8) and eight vertical columns (C1-C8) (Fig 2), and the data were analyzed by the Articulate Assistant software (Articulate Instruments Ltd., Musselburgh, UK).

### Plate classification

An EPG plate was divided into three parts in the mesio-distal direction for detailed analysis (Fig 2).

1. Alveolar part: two rows of the front of the plate (R1 and R2)
2. Palatal part: three rows of the middle of the plate (from R3 to R5)
3. Velar part: three rows of the back of the plate (from R6 to R8)

### Swallowing

Swallowing was evaluated by EPG in all participants. In order to imitate habitual swallowing, Cheng et al. recommended to swallow 3 to 5 ml of water through a straw, wait 10 seconds, and then swallow again [11]. According to this previous study, each participant was seated upright in a chair and swallowed 3 mL of water 5 times with a straw before data taking. They were instructed to keep the head in the Frankfort horizontal plane parallel to the floor, and a sufficient break (10 seconds) was provided between each examination. Each swallowing was also monitored by a video recorder.

### Procedure

All participants were required to set the EPG plate for 1 h before EPG recording to adjust to swallowing with plates in the mouth. The tongue-palatal contact patterns for patients with mandibular prognathism were recorded just before surgery and three months after surgery, according to a previous study that demonstrated that swallowing function can appear to stabilize by three months after orthognathic surgery in patients with mandibular prognathism [12].

### The number of electrodes contact

The total number of electrodes in each frame was regarded as the whole total (WT). Changes in the WT, number of electrodes in contact with the alveolar, palatal, and velar parts of controls and patients with mandibular prognathism were compared pre- and postoperatively.

### Duration of swallowing phase

The period from the complete tongue-palatal contact to the initiation of tongue-palatal separation corresponds to the oral and pharyngeal phase of swallowing. The period from the initiation of tongue-palatal separation to complete separation was recognized as the esophageal phase according to a previous study [10].

### Data analysis

The mean value of WT, the number of electrodes of the alveolar part, palatal part, and velar part at 0.3, 0.2, and 0.1 sec before complete tongue-palatal contact during swallowing, as recorded by the Articulate Assistant, were evaluated. A representative frame of the EPG pattern in controls and mandibular prognathism patients at 0.3, 0.2, and 0.1 sec before complete tongue-palatal contact was also investigated.

### Statistical treatment

Statistical analyses for the WT, the number of electrodes of the alveolar part, palatal part, and velar part as well as the duration of swallowing phase were conducted using analysis of variance and Fisher’s multiple-comparison test. A *p*-value < 0.05 was considered statistically significant. All analyses were performed using StatView 5.0 software.

## Results

### Representative EPG pattern in the controls

**Fig 3:**
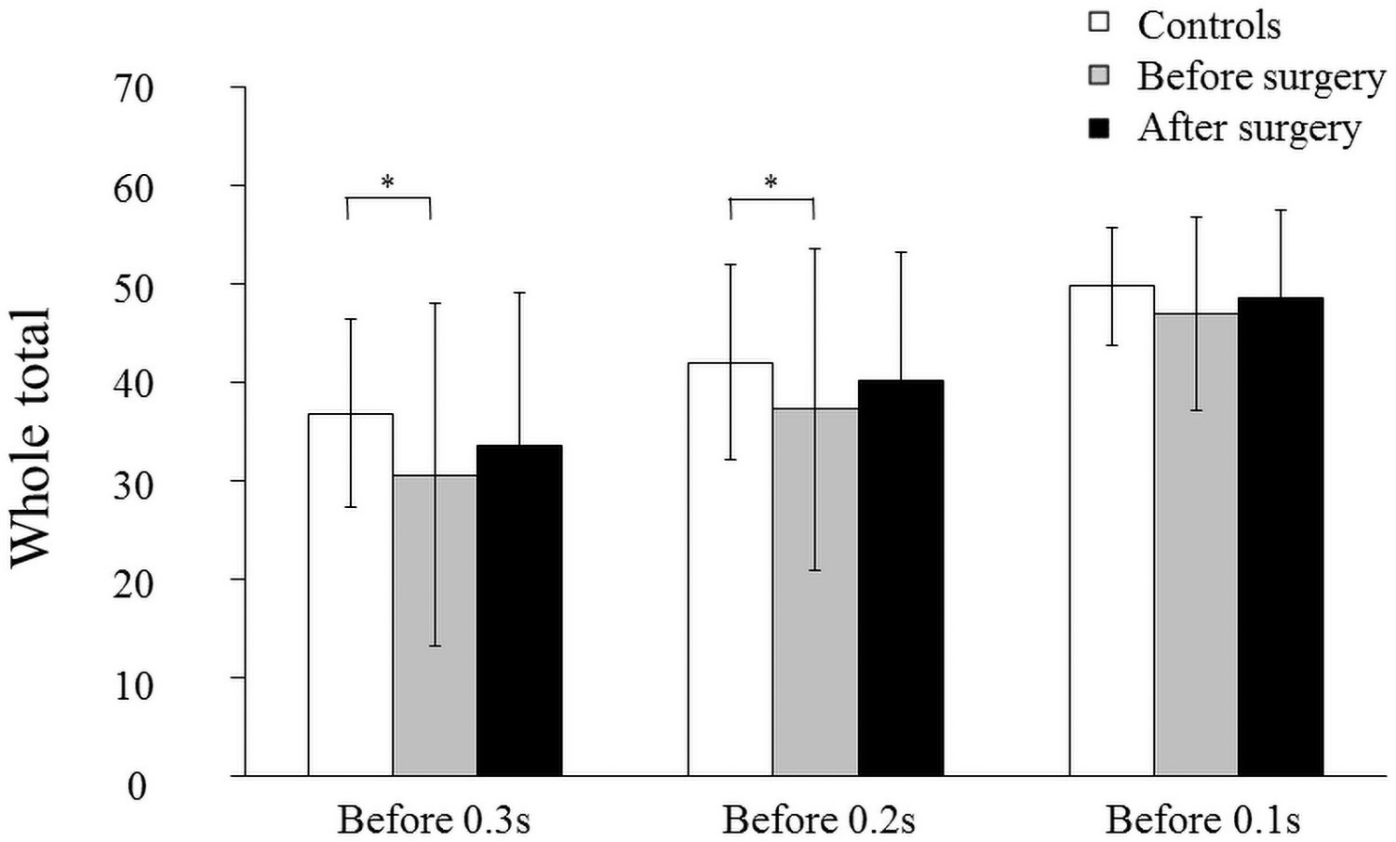
Total number of electrodes contact. Mean and standard deviation in the total number of electrodes of tongue-palatal contact (WT) in the controls, patients before surgery, and after surgery at 0.3, 0.2 and 0.1 seconds before complete tongue-palatal contact (*: *p*<0.05, **: *p*<0.01).

The representative EPG pattern in the controls at 0.3, 0.2, and 0.1 sec before complete tongue-palatal contact are shown in Fig 2. Complete seals in the alveolar part (R1 and R2) were shown at 0.3, 0.2, and 0.1 sec. Most of the electrodes contact was detected in the palatal part (from R3 to R5) at 0.1 sec. WT gradually increased from 0.3 to 0.1 sec (Fig 3).

### Changes in the EPG pattern and WT before and after SSRO in mandibular prognathism patients

No patients complained of swallowing problems after orthognathic surgery. Fig 2 shows the changes in the representative EPG pattern during swallowing of a patient with mandibular prognathism. Preoperatively, incomplete seals in the alveolar part (R1 and R2) were shown at 0.3 sec before complete tongue-palatal contact, however, most electrodes contact was observed in the alveolar part after surgery at 0.3 sec before complete tongue-palatal contact and the EPG patterns became similar to those of controls (Fig 2). WT at 0.3 and 0.2 sec before complete tongue-palatal contact was significantly smaller in patients with mandibular prognathism preoperatively than in the controls (*p* < 0.05). No significant difference was observed in WT among controls, before surgery, and after surgery at 0.1 sec before complete tongue-palatal contact (Fig 3).

### Changes in the number of electrodes contact of alveolar, palatal and velar parts (0.3 sec before complete tongue-palatal contact)

**Fig 4:**
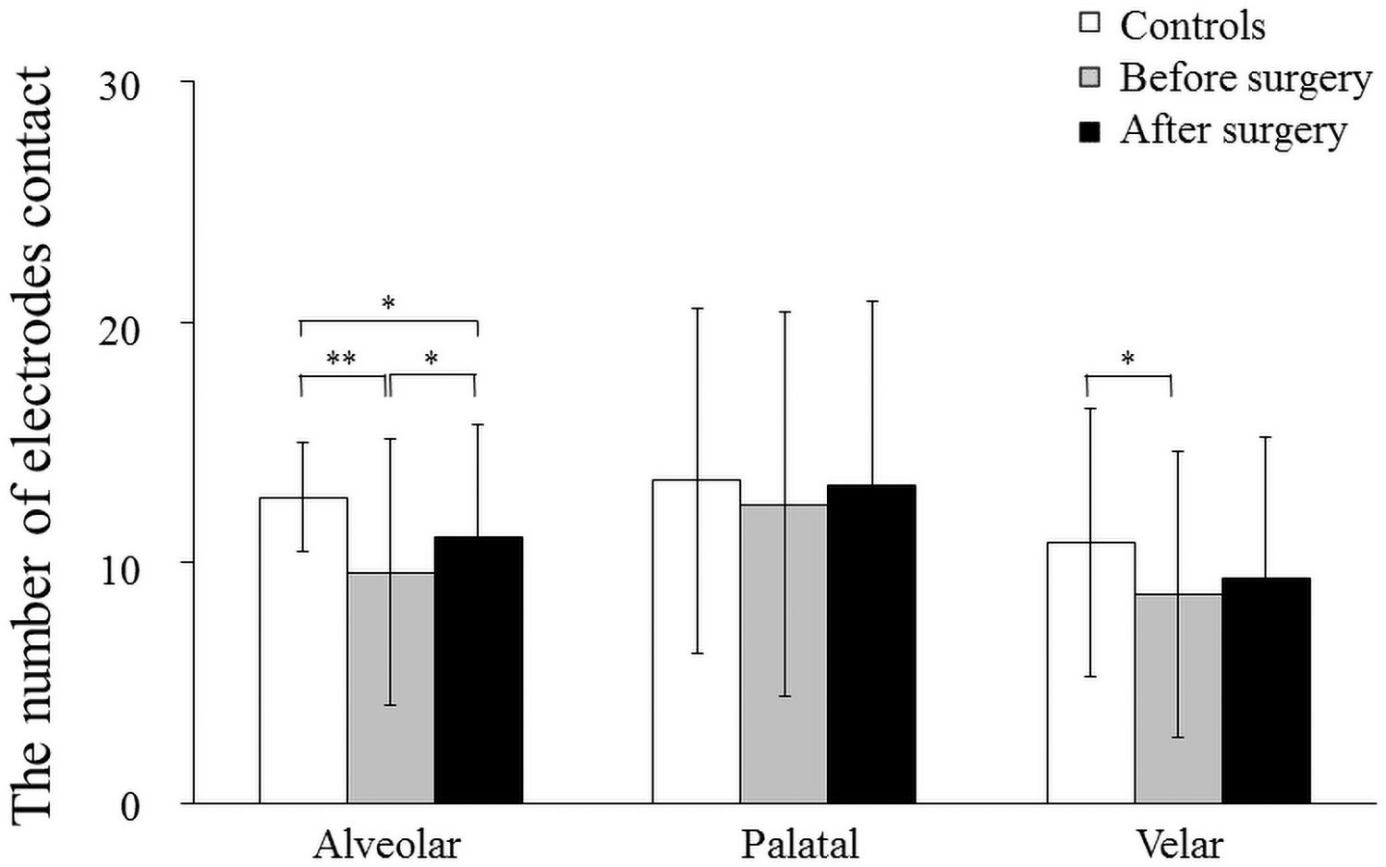
Mean and standard deviation in the number of electrodes contact of alveolar, palatal and velar part in the controls, patients before surgery, and after surgery. 0.3 seconds before complete tongue-palatal contact (*: *p*<0.05, **: *p*<0.01).

The number of electrodes contact in the alveolar part (R1 and R2) at 0.3 sec before complete tongue-palatal contact was significantly higher in the controls than in the patients with mandibular prognathism preoperatively (*p* < 0.01, Fig 4), and a significant increase was observed after surgery compared to that preoperatively (*p* < 0.05, Fig 4). No significant difference was observed among controls, before surgery, and after surgery in the palatal part (from R3 to R5, Fig 4). The number of electrodes in the velar part (from R6 to R8) was significantly higher in the controls than in the patients with mandibular prognathism preoperatively (*p* < 0.05, Fig 4).

### Changes in the number of electrodes contact of alveolar, palatal and velar parts (0.2 sec before complete tongue-palatal contact)

**Fig 5:**
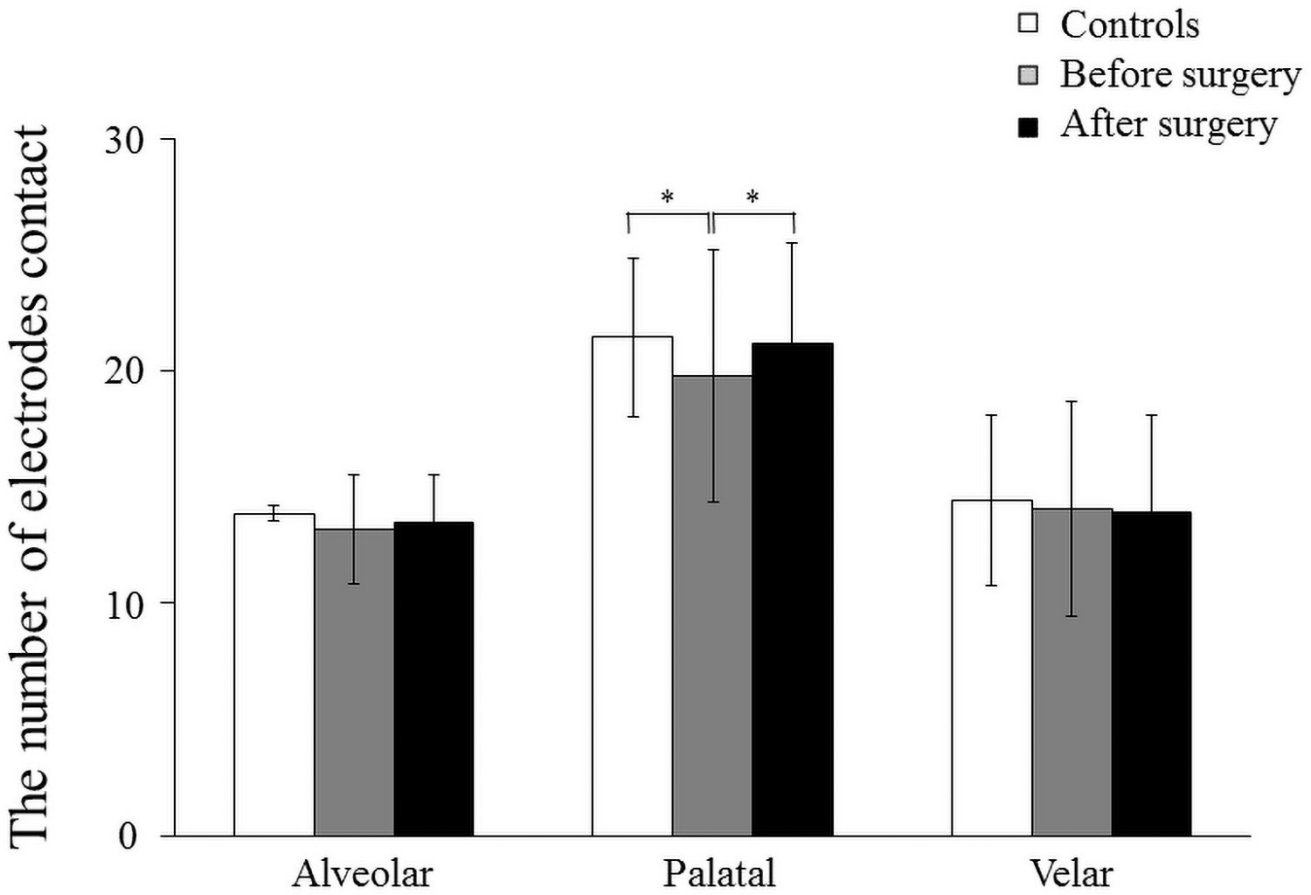
Mean and standard deviation in the number of electrodes contact of alveolar, palatal and velar part in the controls, patients before surgery, and after surgery. 0.2 seconds before complete tongue-palatal contact (*: *p*<0.05, **: *p*<0.01).

At 0.2 sec, the number of electrodes in the alveolar part (R1 and R2) was significantly higher in the controls than in the patients with mandibular prognathism preoperatively (*p* < 0.01, Fig 5). The number of electrodes contact showed a significant increase after surgery compared to that preoperatively in the alveolar part (*p* < 0.05, Fig 5). No significant difference was observed among controls, before surgery, and after surgery in the palatal and velar parts (from R6 to R8, Fig 5).

### Changes in the number of electrodes contact of alveolar, palatal and velar parts (0.1 sec before complete tongue-palatal contact)

**Fig 6:**
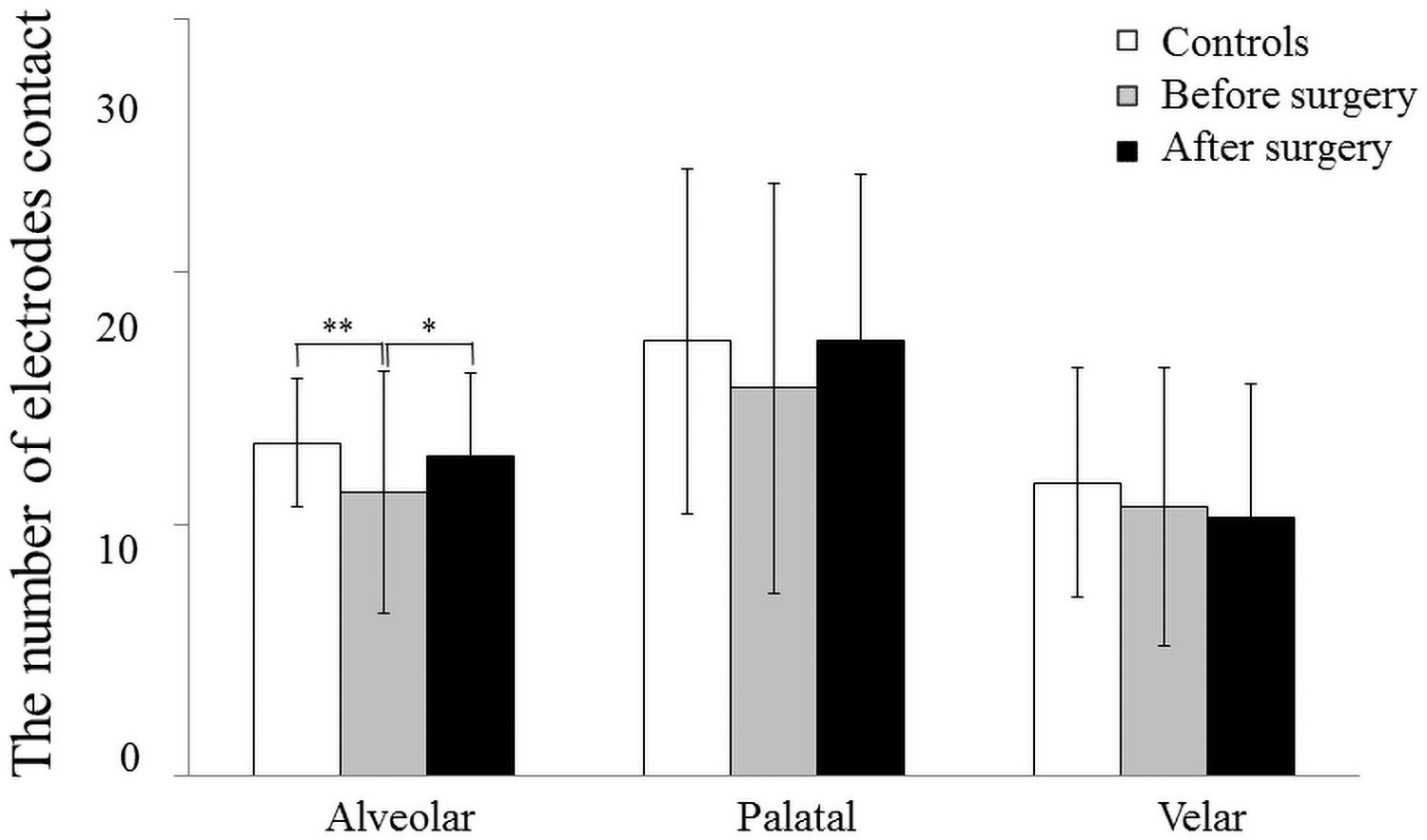
Mean and standard deviation in the number of electrodes contact of alveolar, palatal and velar part in the controls, patients before surgery, and after surgery. 0.3 seconds before complete tongue-palatal contact (*: *p*<0.05).

At 0.1 sec, no significant difference was observed in the number of electrodes contact among controls, before surgery, and after surgery in the alveolar (R1 and R2) and velar parts (from R6 to R8). The number of electrodes contact in the palatal part (from R3 to R5) was significantly higher in the controls and after surgery than in the mandibular prognathism patients preoperatively (*p* < 0.05, Fig 6).

### Duration of swallowing phase

**Fig 7:**
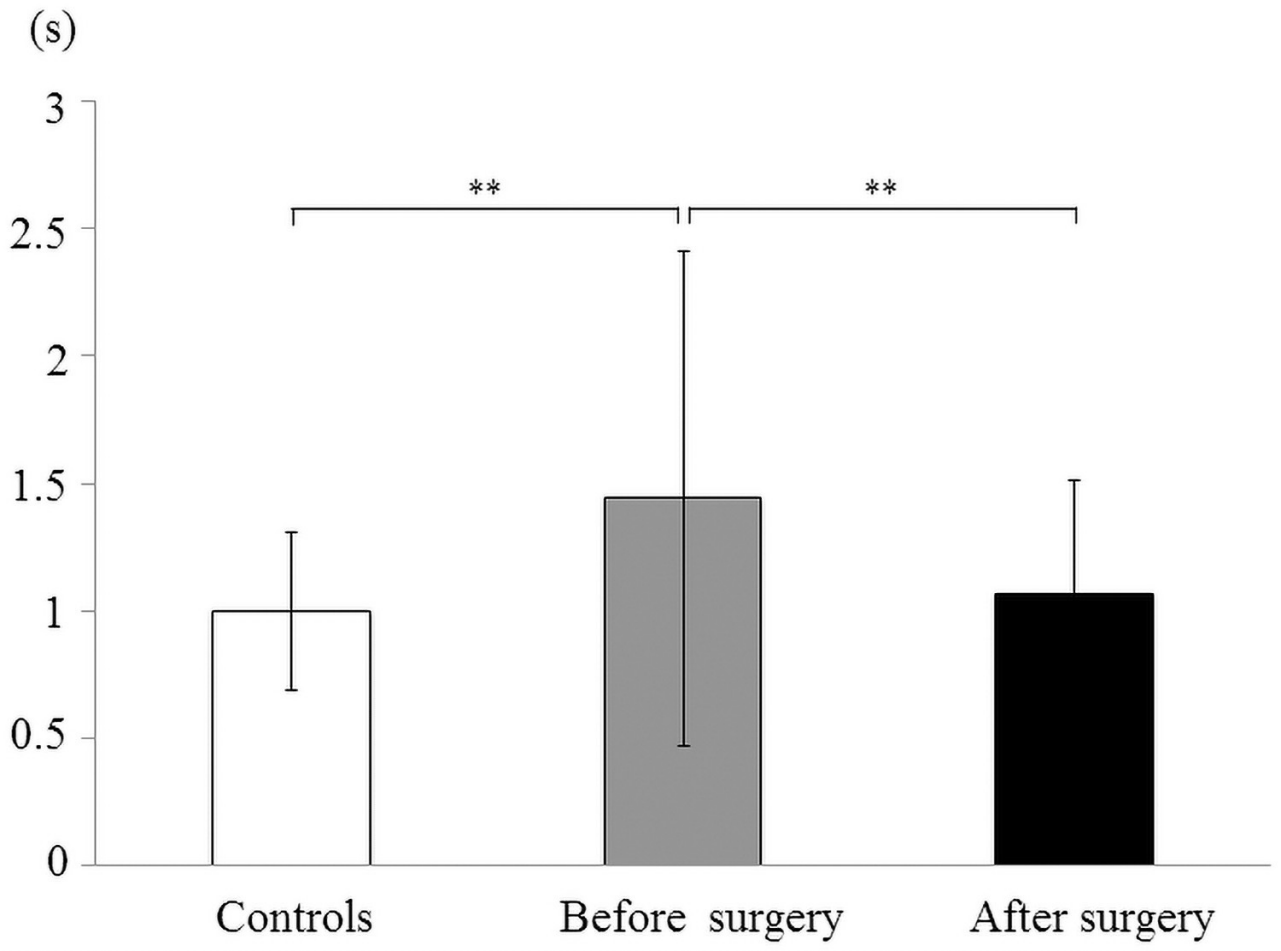
Mean and standard deviation in the duration of swallowing phase in the controls and patients with mandibular prognathism before and after surgery. The duration of the oral and pharyngeal phase (**: *p*<0.01).

**Fig 8:**
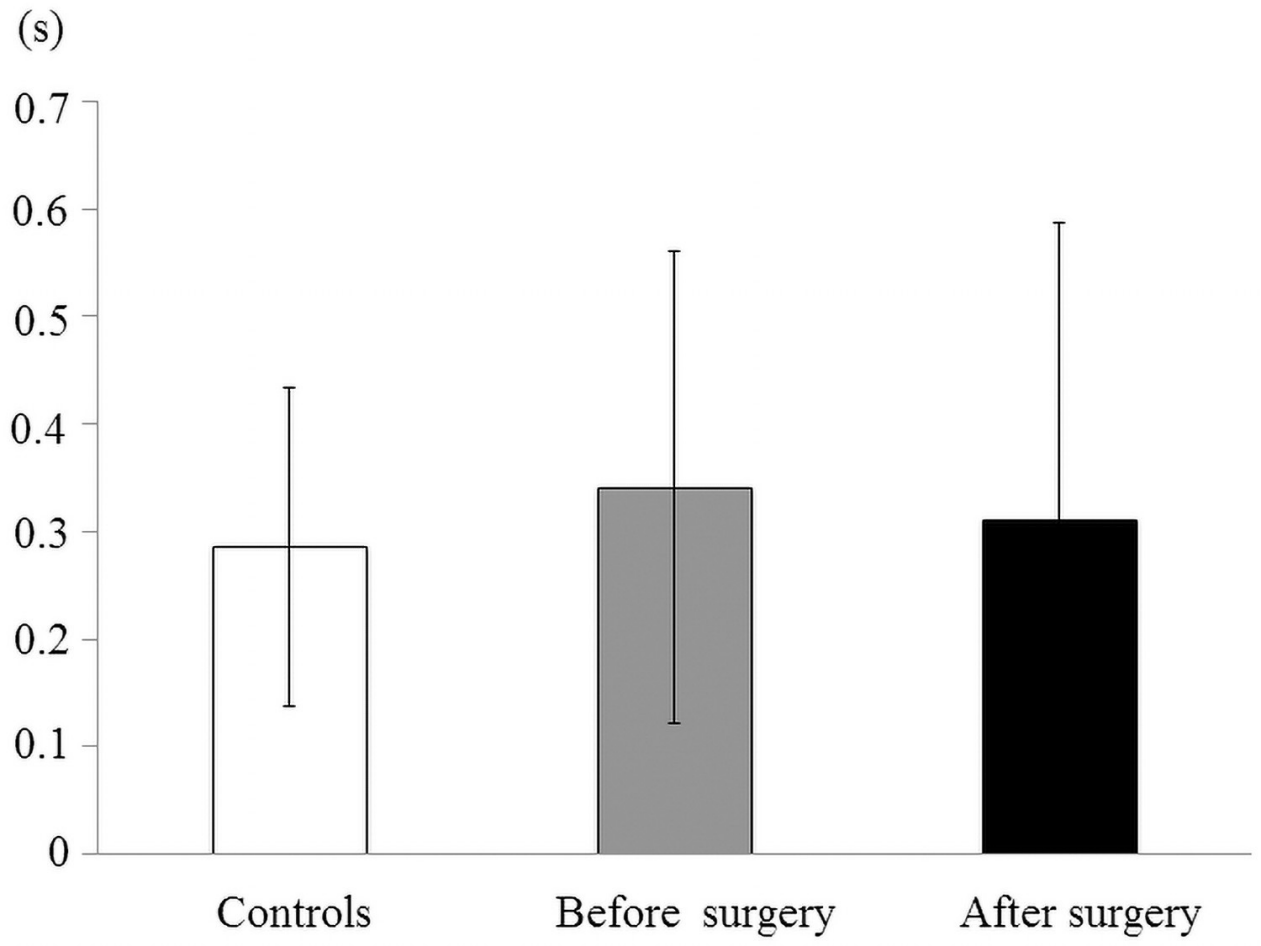
Mean and standard deviation in the duration of swallowing phase in the controls and patients with mandibular prognathism before and after surgery. The duration of the esophageal phase.

The average duration of the oral and pharyngeal phase in the controls and patients with mandibular prognathism pre- and postoperatively are shown in Fig 7. The mean duration in the mandibular prognathism patients preoperatively was significantly higher than that in the controls and the patients after surgery (*p* < 0.01, Fig 7). No significant difference was observed among controls, before surgery, and after surgery in the esophageal phase (Fig 8).

There was no difference in the swallowing pattern including tongue-palatal contact and duration of each phase for the male and female participants.

## Discussion

Swallowing is an essential oral function, and tongue movement is quite important during swallowing. Previous reports have shown that there are correlations between tongue movement during swallowing and maxillofacial morphology [5, 10, 11]. Therefore, it can be expected that swallowing is affected by orthognathic surgery due to changes in the position of the mandible, tongue, and suprahyoid muscles. However, abnormalities and changes in tongue movement during swallowing in patients with mandibular prognathism before and after orthognathic surgery have not been well assessed quantitatively. Fujiki et al. investigated the changes in tongue movement during swallowing following the correction of mandibular prognathism after SSRO by cineradiography. They showed that tongue-palatal contact during swallowing preoperatively was significantly smaller, and the tongue contact could adapt to the corrected oral and maxillofacial morphology after SSRO [13]. Namaki et al. reported that tongue and soft palate movement during swallowing decreased immediately after SSRO in patients with mandibular prognathism, and then resolved three months postoperatively using videofluorography. Therefore, they concluded that SSRO influences tongue movement, and the function of swallowing appears to stabilize by three months after SSRO [12]. Although these previous studies exhibited quantitative data of tongue movement during swallowing, it is difficult to observe tongue-palatal contact on a horizontal projection surface. EPG is a useful device to visualize tongue-palatal contact in the horizontal plane using an acrylic palatal base plate with electrodes [6, 7]. Ichida et al. demonstrated that tongue-palatal contact duration during swallowing was significantly correlated with the measurement items of mandibular rotation and inclination of the upper incisors using EPG [10]. However, this article focused only on patients before orthodontic treatment. Therefore, in the present study, changes in tongue-palatal contact patterns during swallowing before and after orthognathic surgery were evaluated using EPG. In this study, 7 participants underwent Le Fort I osteotomy as well as SSRO. Wakumoto et al. showed that tongue-palatal contact changes before and after orthognathic surgery were similar in the SSRO patient and SSRO with Le Fort I osteotomy patient during articulation using EPG study [14]. Because tongue-palatal contact patterns can be related directly to the changes in the mesio-distal relationship of maxilla and mandible after orthognathic surgery, we included patients who underwent Le Fort I osteotomy as well as SSRO in this study. Another EPG study demonstrated that tongue-palatal contact duration during swallowing with the same persons was consistent and independent of the day of measurement [15]. Therefore, in this study, 5 times data taking for swallowing was performed in the same day.

In the present study, a representative EPG frame at 0.3, 0.2, and 0.1 sec before complete tongue-palatal contact was investigated. Sakaue et al. showed that the tongue pressure waveform in normal participants peaked at 0.2 to 0.3 sec from the first tongue pressure onset during swallowing [4]. According to this findings, we selected the frame of 0.3 sec before complete tongue-palatal contact as a first observation point. Sakaue et al. also reported that the order of tongue pressure production followed the anterior part, peripheral margin, mid-median, and posterior median part in the normal participants, and the maximum tongue pressure value was lower in patients with mandibular prognathism [4]. Fujiki et al. demonstrated that tongue-palatal contact during swallowing before SSRO was significantly smaller and the contact between the tongue tip and lingual part of the anterior teeth showed a decrease compared to that in the controls [13]. The present study showed that complete seals in the alveolar part were shown at 0.3, 0.2, and 0.1 sec before complete tongue-palatal contact and most electrodes contact was detected in the palatal part at 0.1 sec in the controls, although incomplete seals in the alveolar part were shown at 0.3 seconds in patients with mandibular prognathism. These findings suggest that the tongue tip in controls first contacts the cervical area of the anterior teeth, and subsequently the lateral side of the tongue touches the palate. Finally, the middle and posterior parts of the dorsal tongue come into contact with the palate, which can make bolus transport from the anterior to posterior part of the oral cavity during swallowing. However, in patients with mandibular prognathism, it is suspected that a complete seal of the anterior part is difficult due to the front position of the tongue and mandible.

In the present study, the WT was significantly smaller in patients with mandibular prognathism before surgery than in controls at 0.3 and 0.2 sec before complete tongue-palatal contact. A significant increase in the number of electrodes contact of the alveolar part was observed after surgery at 0.3 sec before complete tongue-palatal contact and the EPG patterns became similar to controls, although there was still a significant difference between the controls and the patients after surgery. At 0.2 sec before complete tongue-palatal contact, no significant difference in the number of electrodes contact of the alveolar part was detected between the controls and the patients after surgery, indicating that mandibular setback may cause complete tongue seal of the anterior part and production of intraoral pressure during swallowing. These findings support the results of another study showing that the tongue-palatal contact and tongue tip position changed after surgery, and became similar to those of the controls during swallowing [13]. Therefore, it is clearly suggested that swallowing functions such as tongue-palatal contact and tongue position can improve and adapt to the new oral and maxillofacial circumstance after orthognathic surgery.

The duration of the oral and pharyngeal phase of swallowing was significantly higher in the patients before surgery, but significantly decreased after surgery. There was no statistical difference between the duration of the oral and pharyngeal phase in the controls and the patients after SSRO. This result is similar to that of the study by Cheng et al. [11]. Sakaue et al. also showed that swallowing time was longer in patients with mandibular prognathism than in the control group, and that maximum tongue pressure was lower in the patient group than in the control group [4]. This would mean that prolonged tongue-palatal contact is needed in the oral and pharyngeal phase of swallowing because of the lower tongue pressure. On the other hand, no significant difference was observed among controls, patients before surgery, and patients after surgery in the esophageal phase; this corroborates the results of Fujiki et al. [13]. Therefore, it was suggested that tongue function during swallowing after involuntary movement is not influenced by maxillofacial morphology.

The results of the present study showed that the tongue-palatal contact pattern in patients with mandibular prognathism during swallowing can improve after orthognathic surgery. Since there is a strong relationship between abnormal tongue function and relapses after orthodontic treatment, it is strongly suggested that orthognathic surgery is useful not only for improving skeletal jaw relationships but also for posttreatment occlusal stability. Further clinical investigation will be needed to clarify the aging effect on swallowing in the growing patients with mandibular protrusion. Moreover, various comorbidities such as anomalous cervical carotid artery pathway [16], hypertrophy of the lingual tonsil [17], aneurysmal bone cyst [18] can influence changes in tongue-palatal contact during swallowing. We will try to investigate tongue-palatal contact patterns in patients with dysphagia due to such diseases using EPG in future.

## Conclusions

This study demonstrated that the tongue-palatal contact pattern during swallowing is different, and the duration of the oral and pharyngeal phase is prolonged in patients with mandibular prognathism compared to controls. However, tongue movement can improve after orthognathic surgery. Therefore, it is suggested that morphological changes may induce normal muscle function, such as tongue movement during swallowing.
